# Nuclear factor erythroid 2-related factor 2 is a critical target for the treatment of glucocorticoid-resistant lupus nephritis

**DOI:** 10.1186/s13075-016-1039-5

**Published:** 2016-06-14

**Authors:** Shin Ebihara, Hideaki Tajima, Masao Ono

**Affiliations:** Department of Pathology, Tohoku University Graduate School of Medicine, 2-1 Seiryo, Aoba, Sendai, Miyagi 980-8575 Japan; Department of Clinical laboratory, National Hospital Organization Mito Medical Center, 280 Sakuranosato, Ibaraki, higashi-ibaraki, Ibaraki, 311-3193 Japan

**Keywords:** Nrf2, Dimethyl fumarate, Mesangial cell, Pristane, Lupus nephritis, Glucocorticoid resistance

## Abstract

**Background:**

Dimethyl fumarate (DMF), a nuclear factor erythroid 2-related factor 2 (Nrf2) activator, has been proven effective for the systemic treatment of multiple sclerosis. The aim of this study is to evaluate the anti-inflammatory effects of Nrf2 activators on human renal mesangial cells (HRMCs) and the development of lupus nephritis (LN) in mice.

**Methods:**

To assess Nrf2 activation in vitro, HRMCs were treated with safe doses of Nrf2 activators and prednisolone. The expression levels of Nrf2 and its target genes were measured using quantitative reverse transcription PCR and enzyme-linked immunosorbent assay. The anti-inflammatory effects of these compounds were assessed by measuring tumor necrosis factor alpha-induced cytokine secretion. Experimental LN was induced in female BALB/c mice by a single intraperitoneal injection of pristane. The urine albumin-to-creatinine ratio was measured at 20 weeks after injection. Pathological changes as well as protein and mRNA expression levels were assessed in the kidney obtained at the experimental end point. Oral administration of DMF or prednisolone to these mice was initiated after pristane injection.

**Results:**

Nrf2 activators such as sulforaphane and DMF showed anti-inflammatory effects in HRMCs, whereas glucocorticoid (prednisolone) showed partial effects. Moreover, DMF ameliorated the development of kidney diseases in pristane-induced LN mice, whereas glucocorticoid had no effect.

**Conclusions:**

Nrf2 activators showed stronger anti-inflammatory and organ-protective effects than glucocorticoid in the kidney. Thus, Nrf2 activators are potential therapeutic targets in glucocorticoid-resistant LN in humans.

## Background

Systemic lupus erythematosus (SLE) is an autoimmune disease characterized by inflammation in different organ systems and immunologic abnormalities, such as presence of autoreactive T cells and hyperactive B cells producing autoantibodies [1]. Approximately 25–50 % of SLE patients develop lupus nephritis (LN), which is initiated by the deposition of immune complexes within the renal parenchyma leading to complement activation, mesangial expansion, and induction of inflammatory and fibrotic processes, resulting in glomerulonephritis and progressive renal dysfunction [2]. LN patients are often treated with strong immunosuppressive regimens, including cytotoxic drugs, antimalarial compounds, immunophilin targeting agents, and glucocorticoids [3]. In addition, the effect of these therapeutic medications on LN has been determined by the histologic diagnosis of glomerulonephritis [3]. The treatments can transiently reduce disease activity, but often do not induce remission or prevent end organ damage.

The polymorphisms in the nuclear factor erythroid 2-related factor 2 (Nrf2) genes appear to confer a risk for developing LN [4]. Moreover, Nrf2 deficiency leads to the development of lupus-like autoimmune nephritis in aged female mice [5], indicating that Nrf2 plays a crucial role in the development of LN in both human and mice. Nrf2 is a redox-sensitive transcription factor that regulates the expression of antioxidant response element (ARE)-dependent genes, such as those encoding antioxidant and detoxifying enzymes like heme oxygenase-1 (HO-1) and NAD(P)H:quinone oxidoreductase 1 (NQO1) [6]. Under normal conditions, Nrf2 is retained in the cytoplasm in a “silent” form by its repressor protein, Kelch-like ECH-associated protein 1 (Keap1), which contains a subset of reactive cysteine residues. Oxidative stress or environmental stimuli modifies the cysteine residues of Keap1, thus enabling translocation of Nrf2 to the nucleus where it binds to the ARE [6]. The activation of Nrf2 leads not only to organ-protective effects but also to anti-inflammatory effects by inhibition of nuclear factor (NF)-kB activation. [7]. In a previous study, antioxidant response of Nrf2 and NQO1, which functions in the downstream of Nrf2, was demonstrated in the kidney of patients with LN; however, it was not determined whether the response modified the disease activity or not [8].

Small-molecule compounds such as sulforaphane and dimethyl fumarate (DMF) have been reported as the clinically used Nrf2 activators [9, 10]. Sulforaphane, an organosulfur compound found in broccoli sprouts and other cruciferous vegetables, has potent anti-inflammatory or anticancer activities, and has been clinically used as a dietary supplement [11]. It inhibited proinflammatory cytokine production in vitro in lipopolysaccharide (LPS)-stimulated monocytes [12] and tumor necrosis factor alpha (TNF-α)-stimulated synoviocytes [13]. Furthermore, sulforaphane improved the development of experimental autoimmune encephalomyelitis (EAE) [14] and alleviated type II collagen-induced arthritis [15] and dextran sodium sulfate-induced acute colitis [16] in mice. In contrast, DMF is the main active ingredient of fumaric acid esters (FAEs) and has been approved in Germany as Fumaderm® for the systemic treatment of psoriasis [17]. It demonstrated anti-inflammatory activity both in vitro and in vivo. DMF inhibited proinflammatory cytokine production in TNF-α-stimulated human umbilical vein endothelial cells [18] and LPS/interferon gamma (IFNγ)-stimulated peripheral blood mononuclear cells (PBMCs) [19] by inhibiting either the activation or translocation of NF-kB in vitro. Moreover, DMF ameliorated the development of mouse EAE [10]. The efficacy of oral DMF in the treatment of relapsing–remitting multiple sclerosis (RRMS) has been proven in two phase III studies: DEFINE (Determination of the Efficacy and Safety of Oral Fumarate in relapsing–remitting multiple sclerosis) [20] and CONFIRM (Comparator and an Oral Fumarate in relapsing–remitting multiple sclerosis) [21]. In 2013, DMF was approved by the US Food and Drug Administration (FDA) as first-line therapy for RRMS patients [22]. In-vivo studies in psoriasis patients showed that DMF reduced the development of psoriasis [23], and the clinical efficacy of DMF is being explored in a phase III trial (ClinicalTrials.gov NCT01815723). As already described, the effect of the clinically used Nrf2 activators such as sulforaphane and DMF on the development of various chronic inflammatory diseases has been evaluated. However, there are no reports about the effects of these compounds on the development of human LN.

Here, we investigated the effects of clinically used Nrf2 activators on renal functions both in vitro and in vivo. To assess the in-vitro functions, human renal mesangial cells (HRMCs) were used. Mesangial cells typically have physiological functions including phagocytosis, synthesis of the mesangial extracellular matrix, and control of glomerular hemodynamics via mesangial cell contraction [24]. In response to immunological, toxic, ischemic, or mechanical injury, mesangial cells produce chemokines or cytokines that selectively induce inflammatory cell migration and activation at the site of injury [25]. Pristane-injected mice were treated with DMF, an Nrf2 activator with ensured clinical safety [22], to evaluate its in-vivo effects on the development of LN. Pristane is a natural hydrocarbon oil, also called TMPD (2,6,10,14-tetramethylpentadecane). Pristane-induced LN is associated with hypergammaglobulinemia, lupus-related autoantibody production, and immune complex-mediated glomerulonephritis, and resembles human LN [26]. In this model, Nrf2-deficient mice developed a greater degree of renal damage [8]. We examined possible therapy with Nrf2 activators both in vitro and in vivo, and glucocorticoid, the standard therapy for human LN, was used as the reference compound.

## Methods

### Test compounds

DMF (BG-12), sulforaphane, and prednisolone were purchased from Sigma-Aldrich (St. Louis, MO, USA). For the cellular functional assay, these compounds were dissolved in dimethylsulfoxide (DMSO; Nacalai Tesque, Kyoto, Japan) and stored in aliquots at –20 °C. For the animal model, DMF and prednisolone were suspended in 0.5 % methylcellulose as vehicle.

### Antibodies and flow cytometric analysis

Monoclonal antibodies (mAbs) used were PE-conjugated anti-human CD120b (hTNFR-M1), biotin-conjugated anti-human CD120a (MABTNFR1-B1), and isotype-matching control mAb. Allophycocyanin-conjugated streptavidin was used for staining biotin antibodies. All of these were purchased from BD Biosciences (San Jose, CA, USA). For Fc receptor blocking, we used FcR Blocking Reagent (Miltenyi Biotech, Auburn, CA, USA). Cell surface staining was performed according to standard techniques, and flow cytometry was performed with a FACSCalibur flow cytometer and analyzed using CellQuest™ Pro software (BD Biosciences).

### Cell culture

HRMCs were purchased from ScienCell Research Laboratories (Carlsbad, CA, USA), and maintained in the Mesangial cell medium (ScienCell Research Laboratories) in a humidified atmosphere containing 5 % CO_2_ at 37 °C. HRMCs were used between passages 3 and 6.

### Cellular treatments

HRMCs were plated in 96-well poly-l-lysine-coated plates (BD Biosciences) at a density of 1.5 × 10^4^ cells per well. Nrf2 activators, DMF and sulforaphane, or prednisolone were added to the culture media, and DMSO at a final concentration of 0.1 % in culture medium was used as the vehicle control. To evaluate the cytotoxicity of the compounds, the CellTiter-Glo luminescent cell viability assay was performed according to the manufacturer’s instructions (Promega, Madison, WI, USA). In experiments examining the anti-inflammatory effects of Nrf2 activators or prednisolone on TNF-α-induced monocyte chemoattractant protein-1 (MCP-1) or interleukin-6 (IL-6) production, these compounds were added 1 hour before the addition of TNF-α (R&D Systems, Minneapolis, MN, USA). Cells were cultured for 18 hours, and then the supernatants were collected and stored at –80 °C until they were assayed.

### Measurement of NF-kB or Nrf2 transcriptional activity

The activity of nuclear NF-kB or Nrf2 was determined using TransAM transcriptional factor assaying kits for NF-kB p65 or Nrf2 according to the manufacturer’s instructions (Active Motif, Carlsbad, CA, USA). The nuclear extract kit was from Active Motif. NF-kB p65 protein (Active Motif) or Nrf2 protein (Abnova, Taipei, Taiwan) was used as the standard in each kit.

### Protein quantification

Cells were rinsed with phosphate-buffered saline (PBS) and solubilized in lysis buffer, which was PBS containing 1 mM ethylenediaminetetraacetic acid, 0.5 % Triton X-100, 10 μg/ml leupeptin, 10 μg/ml pepstatin, 100 μM phenylmethanesulfonyl fluoride, and 3 μg/ml aprotinin (all from Sigma-Aldrich). Intracellular HO-1 (R&D Systems) and NQO1 (Cusabio, Wuhan, China) expressions were detected using enzyme-linked immunosorbent assay (ELISA) kits according to the manufacturers’ instructions. The total protein level in the cell lysate was detected using the Pierce™ BCA Protein Assay kit according to the manufacturer’s instructions (Thermo Fisher Scientific, Waltham, MA, USA). The amount of MCP-1 and IL-6 released into the supernatants of the cultured cells was determined using ELISA kits (R&D Systems).

### RNA isolation and real-time PCR

RNAs were isolated from HRMCs using the GenElute mammalian total RNA kit (Sigma-Aldrich). Quantitative reverse transcription PCR (RT-PCR) was performed using the RNA-to-CT 1-step kit (Thermo Fisher Scientific) and the ABI PRISM 7900HT system (Thermo Fisher Scientific). The primers (HO-1: Hs01110250_m1, NQO1: Hs00168547_m1, glucocorticoid receptor alpha (GRα): Hs00230818_m1, GRβ: Hs00354508_m1, glyceraldehyde 3-phosphate dehydrogenase (GAPDH): Hs02758991_g1) were from Thermo Fisher Scientific. The comparative cycle time (CT) was used to normalize transcripts to GAPDH.

### Mice

Female BALB/c mice were obtained from SLC (Shizuoka, Japan) and maintained under specific pathogen-free conditions in the Animal Research Institute of the Tohoku University Graduate School of Medicine, Sendai, Japan. All experiments performed in this study conformed to the ethical guidelines of Tohoku University regarding animal experimentation.

### Determination of the effect of medications on pristane-induced nephritis

Female BALB/c mice were injected intraperitoneally with either 0.5 ml pristane (Sigma-Aldrich) or PBS at 7 weeks of age. The medications were administered daily by oral gavage from the starting day until the experimental end point (for 20 weeks), as described previously [27]. Each treatment group consisted of five to seven mice: vehicle alone, 75 mg/kg body weight (bw) DMF, or 2 mg/kg bw prednisolone. The DMF dose was decided based on the recommended human dose of 480 mg/day for multiple sclerosis (MS) [22]. Conversely, the dose of prednisolone was equal to that in the previous reports [28, 29]. At 20 weeks after injection, urine was collected from these mice for 24 hours using the metabolic cages. After killing the mice under anesthesia, the kidneys were removed and frozen for protein and RNA extraction, or fixed for histology analysis. The concentration of urine albumin was measured by ELISA (Shibayagi, Gunma, Japan) and urine creatinine was measured using Liquitech creatinine PAP II (Roche Diagnostics, Basel, Switzerland) and an automatic analyzer (Hitachi 7180; Hitachi High-Technologies, Tokyo, Japan). The urine albumin-to-creatinine ratio (uACR) was expressed as milligrams of albumin per gram of creatinine.

Serum samples were stored at –80 °C until they were assayed. ELISA kits were used to measure the autoantibody titers, which are anti-double strand (ds) DNA (Shibayagi) and anti-nuclear ribonucleoprotein (nRNP) antibody (Alpha Diagnostic, San Antonio, TX, USA).

The kidneys were homogenized in the lysis buffer as already described and both MCP-1 (R&D Systems) and HO-1 (Takara Bio, Shiga, Japan) levels in the kidney were measured by ELISA kit. The isolation of RNAs from the kidney and the quantitative RT-PCR were performed as already described. Oligonucleotide primers were purchased from Thermo Fisher Scientific: Mm00516005_m1 for HO-1, Mm01253561_m1 for NQO1, Mm00441242_m1 for MCP-1, Mm00446190_m1 for IL-6, Mm00443258_m1 for TNF-α, Mm01256744_m1 for fibronectin (Fn1), Mm01178820_m1 for tumor growth factor beta 1 (TGF-β1), and Mm99999915_g1 for GAPDH.

### Pathological evaluation of LN

The kidneys were fixed in 10 % formalin in 0.01 M phosphate buffer (pH 7.2) and embedded in paraffin wax. Tissue sections were stained with periodic acid–Schiffs (PAS) and used for evaluations. The kidneys were scored by histological criteria as follows: 0 = positive staining in <25 % of glomerular cross-section, 1 = positive staining in 25–50 % of glomerular cross-section, 2 = positive staining in 50–75 % of glomerular cross-section, and 3 = positive staining in >75 % of glomerular cross-section. At least 40 glomeruli per animal were assessed to determine the average score. The scoring systems of histology were determined by a pathologist (MO) and a cytologist (HT) blinded to the treatment.

### Statistical analyses

The significance of average differences among multiple groups was evaluated by the Dunnett’s parametric or nonparametric test. *p* < 0.05 was considered significant.

## Results

### Upregulation of HO-1 and NQO1 expression in HRMCs by Nrf2 activators

**Fig. 1:**
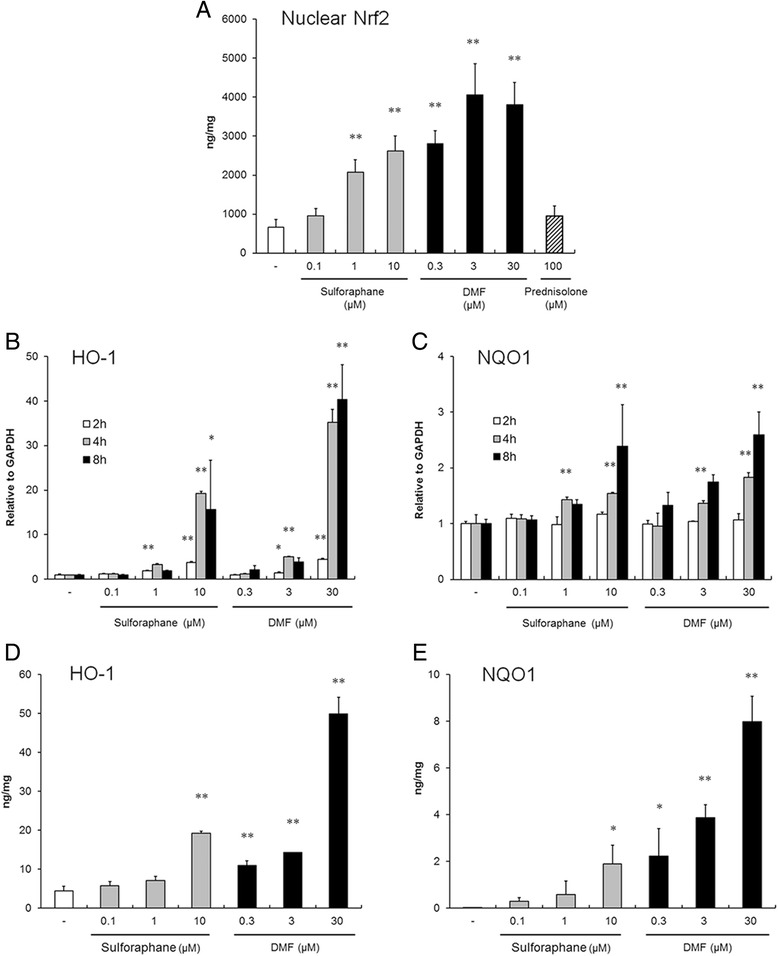
Sulforaphane and DMF induced Nrf2 activation in HRMCs. (**a**) Expression of active Nrf2 in HRMCs. Cells were treated with sulforaphane or DMF, and after 6 hours the nuclear lysates were prepared and the level of active nuclear Nrf2 binding to DNA was measured using ELISA. Results are shown as the ratio of Nrf2 to total protein. (**b**, **c**) mRNA levels of HO-1 and NQO1 expressions. HO-1 (**b**) and NQO1 (**c**) levels were determined using quantitative RT-PCR at 2 (*white bars*), 4 (*gray bars*), and 8 (*black bars*) hours after the stimulation. Data presented are relative mRNA level normalized to GAPDH. (**d**, **e**) HO-1 and NQO1 protein expression. Intracellular HO-1 (**d**) and NQO1 (**e**) concentrations were measured by ELISA 18 hours after the stimulation. Results are shown as the individual protein level to total protein (mean ± SD of three independent experiments). *p < 0.05, **p < 0.01 by Dunnett’s parametric test performed versus DMSO-stimulated group (–). *DMF* dimethyl fumarate, *HO-1* heme oxygenase-1, *NQO1* NAD(P)H:quinone oxidoreductase 1, *Nrf2* nuclear factor erythroid 2-related factor 2

As shown in Fig. 1a, Nrf2 activators such as sulforaphane and DMF induced the binding of Nrf2 to immortalized consensus ARE in HRMCs. Moreover, the Nrf2 activators upregulated the transcription of HO-1 and NQO1, which are the target genes of Nrf2, in a time-dependent and dose-dependent manner at the nontoxic doses (Fig. 1b, c). In HRMCs, Nrf2 activators also significantly increased intracellular HO-1 and NQO1 protein levels 18 hours after the addition of compounds in a dose-dependent manner (Fig. 1d, e).

### Inhibition of the production of MCP-1 and IL-6 by Nrf2 activators

**Fig. 2:**
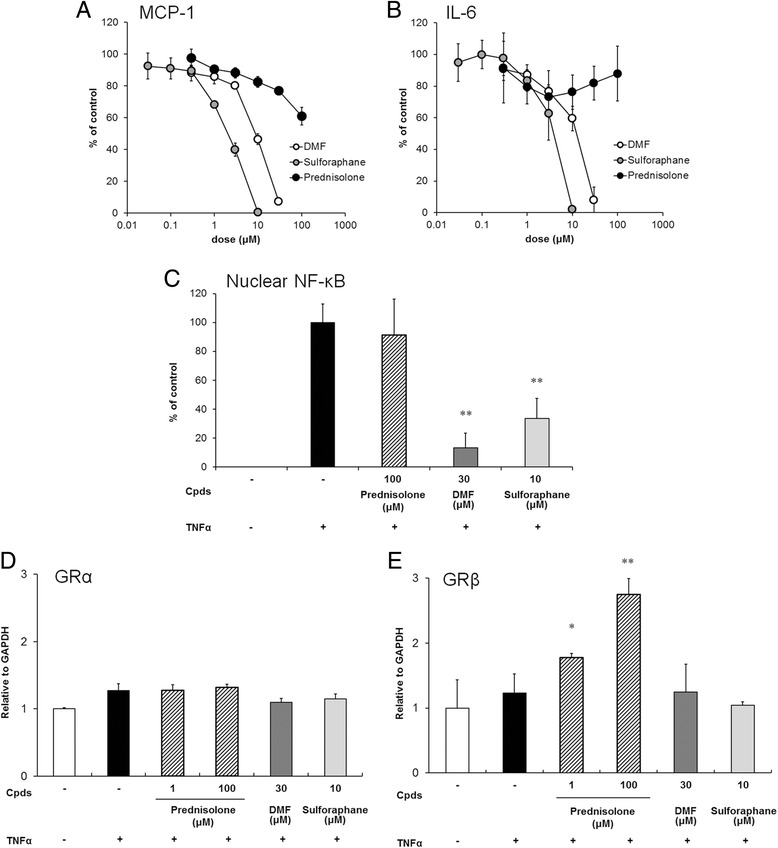
Sulforaphane and DMF showed strong anti-inflammatory effects, whereas prednisolone showed slight effects in HRMCs. (**a**, **b**) Effect of these compounds on inflammatory cytokine production in TNF-α-stimulated HRMCs. Cells were incubated with the nontoxic doses of DMF (*open circles*), sulforaphane (*gray circles*), or prednisolone (*black circles*) prior to TNF-α stimulation. The concentrations of MCP-1 (**a**) and IL-6 (**b**) in the supernatant were determined by ELISA. Results are shown as the % of TNF-α-stimulated (control) groups (mean ± SD of three independent experiments). (**c**) Effect of these compounds on NF-kB activation in TNF-α-stimulated HRMCs. Results are shown as the % of TNF-α-stimulated (control) groups (mean ± SD of three independent experiments). **p < 0.01 by Dunnett’s parametric test performed versus control groups. (**d**, **e**) mRNA levels of GRα and GRβ expression by these compounds exposure under TNF-α stimulation. GRα (**d**) and GRβ (**e**) levels were determined using quantitative RT-PCR 18 hours after the stimulation. Data presented are relative mRNA level normalized to GAPDH. Results are shown as mean ± SD of three independent experiments. *p < 0.05, **p < 0.01 by Dunnett’s parametric test performed versus unstimulated group (–). *DMF* dimethyl fumarate, *GR* glucocorticoid receptor, *IL-6* interleukin-6, *MCP-1* monocyte chemoattractant protein-1, *NF* nuclear factor, *TNF-α* tumor necrosis factor alpha

FACS analysis revealed the expression of CD120a (TNFR1) and CD120b (TNFR2) on the cell surface of HRMCs (data not shown). The HRMCs secreted MCP-1 or IL-6 after TNF-α stimulation in a dose-dependent manner (data not shown). To investigate the anti-inflammatory effects of the test compounds, the inhibition of MCP-1 and IL-6 production in TNF-α-stimulated HRMCs was examined. As shown in Fig. 2a, b, Nrf2 activators such as sulforaphane and DMF inhibited the production of MCP-1 and IL-6 in a dose-dependent manner. On the contrary, surprisingly, prednisolone showed very less inhibition even at the maximum dose of 100 μM (Fig. 2a, b). The inhibitory concentrations (IC_50_) of test compounds for MCP-1 and IL-6 production are presented in Table 1. To determine whether NF-kB is involved in Nrf2 pathway, the effects of Nrf2 activators on TNF-α-induced NF-kB DNA binding were examined in terms of NF-kB activation. Although the Nrf2 activators, sulforaphane and DMF, suppressed TNF-α-induced p65 DNA binding, prednisolone did not suppress the binding even at the maximum nontoxic dose (Fig. 2c). Taken together, these results suggest that activating the Nrf2 pathway with sulforaphane or DMF inhibited the TNF-α-mediated proinflammatory responses in HRMCs.

**Table 1:** Effects of Nrf2 activators and prednisolone on MCP-1 and IL-6 production in TNF-α-stimulated HRMCs^a^

IC_50_ (μM)	MCP-1	IL-6
DMF	8.8	12.2
Sulforaphane	2.0	3.9
Prednisolone	>100	>100

^a^Concentrations of 50 % inhibition (*IC*_*50*_) of MCP-1 and IL-6 production were calculated from sigmoidal inhibition curves presented in Fig. 3. The IC_50_ values were obtained from three individuals

*DMF* dimethyl fumarate, *HRMC* human renal mesangial cell, *IL-6* interleukin-6, *MCP-1* monocyte chemoattractant protein-1, *Nrf2* nuclear factor erythroid 2-related factor 2, *TNF-α* tumor necrosis factor alpha

To investigate the cause of glucocorticoid insensitivity, the alteration of mRNA expression of GRα (Fig. 2d) and GRβ (Fig. 2e) by prednisolone treatment under TNF-α stimulation was examined. GRα functions as a glucocorticoid-dependent transcription factor, whereas GRβ acts as a dominant negative inhibitor of GRα in human cells [30]. The exposure of HRMCs to prednisolone upregulated GRβ in a dose-dependent manner (Fig. 2e). On the contrary, GRα mRNA expression did not appear to be altered significantly in the cells (Fig. 2d), suggesting that the increase in GRβ expression contributes to the glucocorticoid insensitivity in HRMCs.

### Amelioration of kidney disease as manifested by reduced proteinuria and glomerulonephritis in pristane-injected mice treated with DMF

**Fig. 3:**
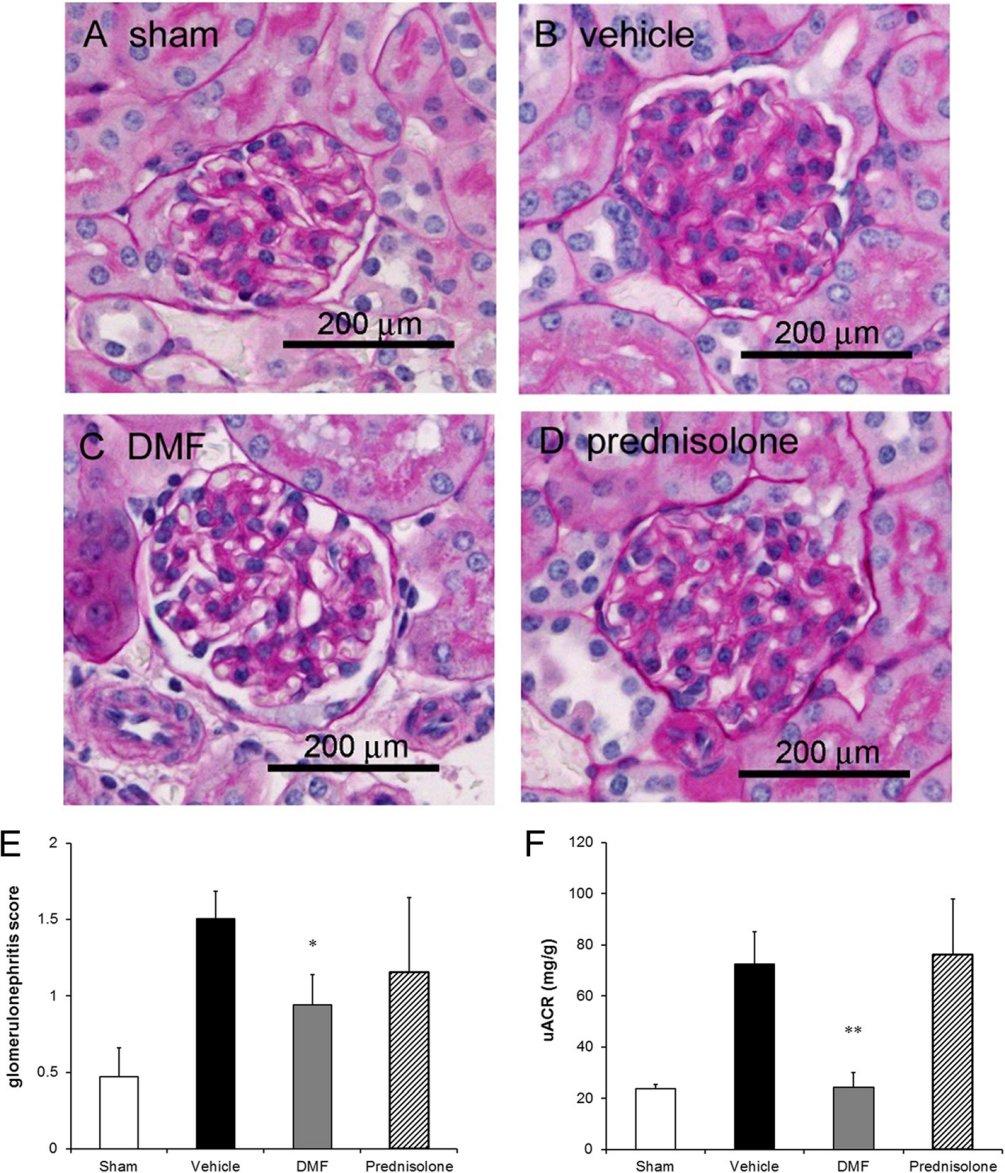
Prevention of development of kidney diseases upon administration of DMF in pristane-injected mice. (**a**–**d**) Representative microphotograms of renal tissue obtained from PBS/0.5 % methylcellulose (sham)-treated (**a**), pristane/0.5 % methylcellulose (vehicle)-treated (**b**), pristane/DMF-treated (**c**), and pristane/prednisolone-treated (**d**) mice (PAS staining). Scale bar, 200 μm. (**e**) Glomerulonephritis score of pristane-induced LN of the four groups of mice at the experimental end point. *p < 0.05 by Dunnett’s nonparametric test performed versus sham-treated group. (**f**) At 20 weeks after pristane injection, the urine albumin-to-creatinine ratio (uACR) was measured. **p < 0.01 by Dunnett’s parametric test performed versus sham-treated group. *n* = 5–7 animals per group. *DMF* dimethyl fumarate

To explore the effect of Nrf2 activator on the progression of LN, a pristane-induced murine model was used. Female BALB/c mice were treated daily by oral gavage with either vehicle only, 75 mg/kg bw DMF, or 2 mg/kg bw prednisolone after intraperitoneal injection of pristane. It has been established that, after oral intake, one part of DMF is converted in the small intestinal mucosa to monomethyl fumarate (MMF), and the other part is absorbed by the tissues followed by conjugation to glutathione [31]. Thus, both DMF and MMF play the role of Nrf2 activators in vivo following DMF treatment, as described previously [32]. The DMF dose was decided based on the recommended human dose for MS, which is calculated using plasma MMF exposure (area under the blood concentration–time curve) according to that reported by Biogen Inc. [21], whereas the dose of prednisolone was equal to that in the previous reports [28, 29]. All of the vehicle-administered mice (*n* = 7) developed LN to varied extent. In contrast, none of the DMF-treated mice (*n* = 7) presented overt LN. As shown in Fig. 3a, b, the kidneys obtained from these mice displayed histopathological characteristics of class II LN with mesangial hypercellularity and matrix expansion at the experimental end point. None of the glomeruli met histopathological conditions for class III or class IV LN. These findings largely follow the observations reported previously in pristane-immunized mice [26]. Microscopic examination revealed that DMF exhibited a remarkable suppression of renal disease (Fig. 3a–e). Unexpectedly, the treatment of prednisolone had no effect on the pathological changes (Fig. 3a–e). Pristane increased uACR, an indicator of kidney vascular permeability, at 20 weeks after the pristane injection (Fig. 3f). DMF ameliorated the renal functions, whereas prednisolone did not influence the pathologies (Fig. 3f). Taken together, DMF improved the kidney disease as manifested by reduced proteinuria and histopathological analysis.

### Inhibition of the production of serum autoantibodies and renal inflammatory cytokines in pristane-induced LN treated with DMF

**Fig. 4:**
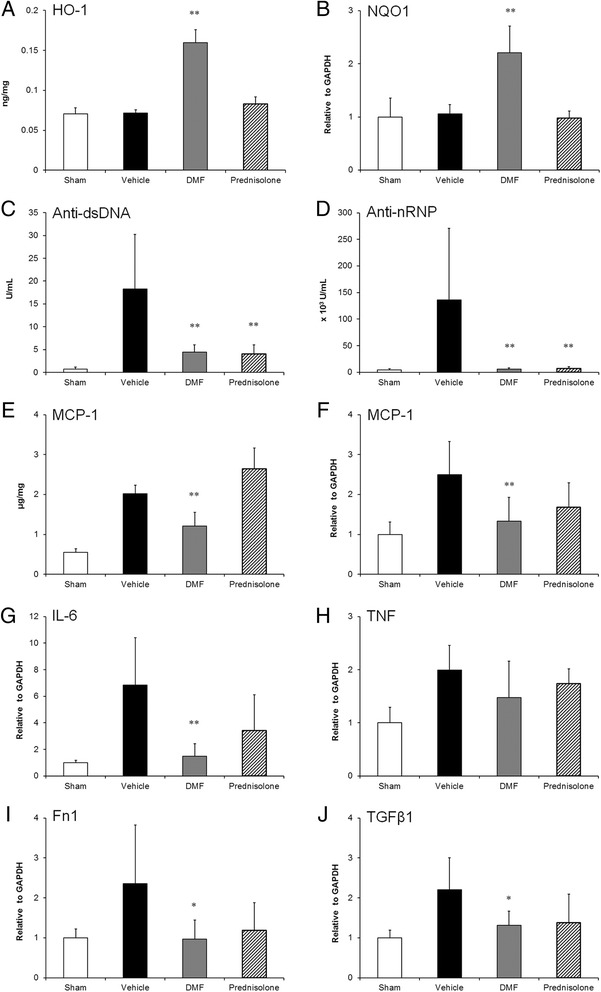
Effects of DMF and prednisolone on inflammation responses in the serum and the kidney. (**a**) Protein level of HO-1 in kidney measured by ELISA. (**b**) mRNA level of NQO1 measured by quantitative RT-PCR. Data presented are relative mRNA level normalized to GAPDH. (**c**, **d**) ELISA kits were used to measure the total immunoglobulins of anti-dsDNA (**c**) and anti-nRNP (**d**) in sera. (**e**) Protein level of MCP-1 in kidney measured by ELISA. (**f**–**j**) MCP-1 (**f**), IL-6 (**g**), TNF-α (**h**), Fn1 (**i**), and TGF-β1 (**j**) in the kidney measured by quantitative RT-PCR. *p < 0.05, **p < 0.01 by Dunnett’s parametric test performed versus sham-treated group. *n* = 5–7 animals per group. *DMF* dimethyl fumarate, *Fn1* fibronectin, *HO-1* heme oxygenase-1, *IL-6* interleukin-6, *MCP-1* monocyte chemoattractant protein-1, *NQO1* NAD(P)H:quinone oxidoreductase 1, *TNF-α* tumor necrosis factor alpha, *TGFβ1* tumor growth factor beta 1

To explore the in-vivo effects of DMF as an Nrf2 activator, the expressions of HO-1 and NQO1 were examined in the kidneys of pristane-injected mice. At the experiment end point, both HO-1 protein (Fig. 4a) and NQO1 transcript (Fig. 4b) significantly increased after DMF treatment within the kidneys of pristane-immunized mice. However, the mRNA expression of HO-1 was not detected (data not shown). Since the kidney samples of these mice were obtained 24 hours after the last administration of the compounds, it is possible that the HO-1 transcription had already finished. Pristane increased serum anti-dsDNA (Fig. 4c) and anti-nRNP (Fig. 4d) antibodies in mice at 20 weeks post injection, as described previously [26]. Moreover, not only DMF but also prednisolone significantly decreased the serum levels of anti-dsDNA (Fig. 4c) and anti-nRNP (Fig. 4d) antibodies compared with the sham group. The results of protein or mRNA quantification analysis performed using the whole kidney showed significant increase in the expression of MCP-1 (Fig. 4e, f) and IL-6 (Fig. 4g), which were produced in various cells including renal cells and infiltrating inflammatory cells, in pristane-injected mice. Further, the analysis of the kidney samples from the mice treated with DMF demonstrated a significant decrease in the expressions of MCP-1 (Fig. 4e, f) and IL-6 (Fig. 4g). In contrast, prednisolone treatment did not change the production in the kidney (Fig. 4e–g). In addition, the TNF-α level was not influenced by administration of DMF or prednisolone (Fig. 4h). Fibronectin is a component of the glomerular basement membrane and mesangial matrix, and accumulates along with other extracellular matrix constituents in proliferative LN. The fibronectin expression of the kidney significantly increased after pristane injection, as described previously (Fig. 4i) [8]. However, the expression was significantly inhibited by treatment with DMF but not prednisolone (Fig. 4i), as supported by the histopathological analysis. TGF-β1, which is a key factor in fibrosis, also increased in pristane-induced LN (Fig. 4i). Nevertheless, administration of DMF significantly decreased the expression (Fig. 4j). On the contrary, prednisolone treatment did not decrease the expression (Fig. 4j). Taken together, DMF protected against renal damage in murine LN by both altering the organ-protection pathway and inhibiting the renal inflammation.

## Discussion

Several reports have shown that glucocorticoid can effectively inhibit proinflammatory cytokine or chemokine production in human immune cells, including PBMCs or monocytes [33, 34]. However, glucocorticoid-insensitive NF-kB activation and proinflammatory cytokine production have been demonstrated in some nonimmune human cells, for example brain cells [35] and airway epithelial cells [36]. Consistent with the described reports, glucocorticoid (prednisolone) showed only a small effect on IL-6 (Fig. 3a) and MCP-1 (Fig. 3b) production in TNF-α-stimulated HRMCs in the present study. The association of glucocorticoid residence and expression of GRβ, which is a dominant negative inhibitor of GRα, have been clinically studied in immune-mediated diseases such as rheumatoid arthritis [37] and ulcerative colitis [38]. The present study revealed that the exposure of mesangial cells to glucocorticoid under inflammatory stimulation increased GRβ expression (Fig. 2e). Taken together, the reaction of human mesangial cells in inflammatory response may be associated with glucocorticoid-insensitive renal diseases.

Among the many Nrf2 activators, we selected DMF that has ensured clinical safety and evaluated its effect in an in-vivo model. Another Nrf2 activator, bardoxolone methyl, was developed for the treatment of chronic kidney diseases and type II diabetes mellitus. However, the phase III BEACON (Bardoxolone Methyl Evaluation in Patients with Chronic Kidney Disease and Type 2 Diabetes Mellitus: the Occurrence of Renal Events) trial was terminated owing to severe renal dysfunction and cardiovascular failure [39]. The safety data observed with DMF suggest that bardoxolone methyl’s side effects may be compound specific rather than target related. Bardoxolone methyl as an Nrf2 activator was expected to be a new therapeutic approach for renal diseases. In fact, bardoxolone methyl has ameliorated kidney disease in models of ischemic acute kidney injury [40], and furthermore in genetic lupus mice [41]. The other compounds which have been shown to activate Nrf2, such as epigallocatechin-3-gallate [42] or hemin [43], have also suppressed renal disease in spontaneous lupus mice, such as NZB/W F1 or MRL/*lpr* mice.

In the present study, we used pristane-induced LN, which resembles the pathology of human LN [26], as the animal model. Pristane treatment of the peritoneal cavity induced apoptosis of peritoneal cells [44] leading to the production of autoantibodies, resulting in the development of LN. Extranodal lymphoid tissue (also called tertiary lymphoid neogenesis) formed in the peritoneal cavity [45] and was indicative of the inflammatory lesion in this pristane-treated model as well as in human LN with tubulointerstitial inflammation [46]. It has been indicated that chronic inflammatory condition leading to lymphoid neogenesis is associated with autoantibody production [47].

There are no histopathological studies on fibrosis of the kidney in pristane-induced LN. In the present study, the upregulation of TGF-β1 expression in the kidney of pristane-injected mice was detected at the experimental end point (Fig. 4j), suggesting that the fibrosis in the kidney of this model may be caused after LN onset. Pristane caused arthritis in some strains of mice with characteristics such as synovial hyperplasia, periostitis, and marginal erosions [48]. The pristane-induced arthritis was strongly inhibited by treatment with prednisolone (2 mg/kg bw) [49]. However, the present study revealed that treatment with the same dose of prednisolone did not inhibit the development of pristane-induced LN (Figs. 3 and 4), suggesting that the difference in the effects may be dependent on organ specificity, including renal cells such as mesangial cells or other nonimmune cells. Interestingly, different from the strongly glucocorticoid-sensitive LN in NZB/W F1 [28] or MRL/*lpr* [29] mice, pristane-induced LN was observed to be glucocorticoid-insensitive LN (Fig. 3). Our present findings suggest upregulation of GRβ expression, which remains to be identified in mice LN models, in nonimmune cells such as mesangial cells.

Although prednisolone inhibited autoantibody production, kidney disease was not improved in the mice (Figs. 3 and 4). The clinical trial in human LN has also demonstrated that the elimination of autoantibodies by plasmapheresis was unsuccessful [50]. Furthermore, the results of another phase III clinical trial—the LUNAR (Lupus Nephritis Assessment with Rituximab) study—revealed that the treatment of rituximab to deplete B cells in LN (class III or class IV) has not resulted in more efficient clinical improvement compared with conventional therapy, despite the reduction of serum anti-dsDNA antibody levels [51]. Taken together, it is evident that it is very important for the therapy against LN to directly prevent the kidney disease and not inhibit the autoantibody production including the glomerular deposition of immune complexes.

In conclusion, Nrf2 activators had not only anti-inflammatory effects but also organ-protective effects on LN pathology. In other words, targeted Nrf2 drugs may be more effective than the currently used immunosuppressants. The results of two phase III studies about DMF revealed that there were no cases of renal adverse events [20, 21] and hence DMF is expected to be a new therapeutic approach for LN. Thus, Nrf2 activators such as DMF, which do not cause serious renal adverse events, are promising alternatives to glucocorticoids for treating excessive immunological activation in damaged or inflamed kidney as well as other inflammatory diseases, such as MS and psoriasis. Our findings provide useful information for the possible application of Nrf2 activators for the treatment of glucocorticoid-resistant LN patients.

## Conclusions

In this study, we found that Nrf2 activators showed anti-inflammatory and organ-protective effects in the kidney. Glucocorticoid (prednisolone) showed only a small effect on proinflammatory cytokine production in TNF-α-stimulated HRMCs, whereas Nrf2 activators showed strong anti-inflammatory effects in the cells. Furthermore, the Nrf2 activator DMF ameliorated the development of kidney diseases in pristane-induced LN mice, whereas glucocorticoid did not have any effect. Thus, Nrf2 activators are potential therapeutic targets in glucocorticoid-resistant LN in humans.

## Abbreviations

ARE, antioxidant response element; BEACON, Bardoxolone Methyl Evaluation in Patients with Chronic Kidney Disease and Type 2 Diabetes Mellitus, the Occurrence of Renal Events; bw, body weight; CONFIRM, Comparator and an Oral Fumarate in Relapsing–Remitting Multiple Sclerosis; DEFINE, Determination of the Efficacy and Safety of Oral Fumarate in Relapsing–Remitting Multiple Sclerosis; DMF, dimethyl fumarate; DMSO, dimethylsulfoxide; ds, double strand; EAE, experimental autoimmune encephalomyelitis; ELISA, enzyme-linked immunosorbent assay; FAE, fumaric acid ester; FDA, Food and Drug Administration; GAPDH, glyceraldehyde 3-phosphate dehydrogenase; GR, glucocorticoid receptor; HO-1, heme oxygenase-1; HRMC, human renal mesangial cell; IFNγ, interferon gamma; IL-6, interleukin-6; Keap1, Kelch-like ECH-associated protein 1; LN, lupus nephritis; LPS, lipopolysaccharide; LUNAR, Lupus Nephritis Assessment with Rituximab; mAb, monoclonal antibody; MCP-1, monocyte chemoattractant protein-1; MMF, monomethyl fumarate; MS, multiple sclerosis; NF, nuclear factor; NQO1, NAD(P)H:quinone oxidoreductase 1; Nrf2, nuclear factor erythroid 2-related factor 2; nRNP, nuclear ribonucleoprotein; PAS, periodic acid–Schiffs; PBMC, peripheral blood mononuclear cell; PBS, phosphate-buffered saline; RRMS, relapsing–remitting multiple sclerosis; RT-PCR, reverse transcription PCR; SLE, systemic lupus erythematosus; TGF-β1, tumor growth factor beta 1; TMPD, 2,6,10,14-tetramethylpentadecane; TNF-α, tumor necrosis factor alpha; uACR, urine albumin-to-creatinine ratio

## References

[1] Rahman A, Isenberg DA (2008). Systemic lupus erythematosus. N Engl J Med..

[2] Cameron JS (1999). Lupus nephritis. J Am Soc Nephrol..

[3] Hahn BH, McMahon MA, Wilkinson A, Wallace WD, Daikh DI, Fitzgerald JD (2012). American College of Rheumatology guidelines for screening, treatment, and management of lupus nephritis. Arthritis Care Res (Hoboken).

[4] Córdova EJ, Velázquez-Cruz R, Centeno F, Baca V, Orozco L (2010). The NRF2 gene variant, -653G/A, is associated with nephritis in childhood-onset systemic lupus erythematosus. Lupus..

[5] Yoh K, Itoh K, Enomoto A, Hirayama A, Yamaguchi N, Kobayashi M (2001). Nrf2-deficient female mice develop lupus-like autoimmune nephritis. Kidney Int..

[6] Suzuki T, Motohashi H, Yamamoto M (2013). Toward clinical application of the Keap1-Nrf2 pathway. Trends Pharmacol Sci..

[7] Wakabayashi N, Slocum SL, Skoko JJ, Shin S, Kensler TW (2010). When NRF2 talks, who's listening?. Antioxid Redox Signal..

[8] Jiang T, Tian F, Zheng H, Whitman SA, Lin Y, Zhang Z (2014). Nrf2 suppresses lupus nephritis through inhibition of oxidative injury and the NF-kB-mediated inflammatory response. Kidney Int..

[9] McMahon M, Itoh K, Yamamoto M, Hayes JD (2003). Keap1-dependent proteasomal degradation of transcription factor Nrf2 contributes to the negative regulation of antioxidant response element-driven gene expression. J Biol Chem..

[10] Linker RA, Lee DH, Ryan S, van Dam AM, Conrad R, Bista P (2011). Fumaric acid esters exert neuroprotective effects in neuroinflammation via activation of the Nrf2 antioxidant pathway. Brain..

[11] Houghton CA, Fassett RG, Coombes JS (2013). Sulforaphane: translational research from laboratory bench to clinic. Nutr Rev..

[12] Rushworth SA, MacEwan DJ, O'Connell MA (2008). Lipopolysaccharide-induced expression of NAD(P)H:quinone oxidoreductase 1 and heme oxygenase-1 protects against excessive inflammatory responses in human monocytes. J Immunol..

[13] Fragoulis A, Laufs J, Müller S, Soppa U, Siegl S, Reiss LK (2012). Sulforaphane has opposing effects on TNF-alpha stimulated and unstimulated synoviocytes. Arthritis Res Ther..

[14] Li B, Cui W, Liu J, Li R, Liu Q, Xie XH (2013). Sulforaphane ameliorates the development of experimental autoimmune encephalomyelitis by antagonizing oxidative stress and Th17-related inflammation in mice. Exp Neurol..

[15] Kong JS, Yoo SA, Kim HS, Kim HA, Yea K, Ryu SH (2010). Inhibition of synovial hyperplasia, rheumatoid T cell activation, and experimental arthritis in mice by sulforaphane, a naturally occurring isothiocyanate. Arthritis Rheum..

[16] Wagner AE, Will O, Sturm C, Lipinski S, Rosenstiel P, Rimbach G (2013). DSS-induced acute colitis in C57BL/6 mice is mitigated by sulforaphane pre-treatment. J Nutr Biochem..

[17] Mrowietz U, Asadullah K (2005). Dimethylfumarate for psoriasis: more than a dietary curiosity. Trends Mol Med..

[18] Loewe R, Holnthoner W, Gröger M, Pillinger M, Gruber F, Mechtcheriakova D (2002). Dimethylfumarate inhibits TNF-induced nuclear entry of NF-kappa B/p65 in human endothelial cells. J Immunol..

[19] Lehmann JC, Listopad JJ, Rentzsch CU, Igney FH, von Bonin A, Hennekes HH (2007). Dimethylfumarate induces immunosuppression via glutathione depletion and subsequent induction of heme oxygenase 1. J Invest Dermatol..

[20] Gold R, Kappos L, Arnold DL, Bar-Or A, Giovannoni G, Selmaj K (2012). Placebo-controlled phase 3 study of oral BG-12 for relapsing multiple sclerosis. N Engl J Med.

[21] Fox RJ, Miller DH, Phillips JT, Hutchinson M, Havrdova E, Kita M (2012). Placebo-controlled phase 3 study of oral BG-12 or glatiramer in multiple sclerosis. N Engl J Med.

[22] Biogen Inc (2013). TECFIDERA Full Prescribing Information.

[23] Bovenschen HJ, Langewouters AM, van de Kerkhof PC (2010). Dimethylfumarate for psoriasis: Pronounced effects on lesional T-cell subsets, epidermal proliferation and differentiation, but not on natural killer T cells in immunohistochemical study. Am J Clin Dermatol..

[24] Rodriguez-Barbero A, L'Azou B, Cambar J, López-Novoa JM (2000). Potential use of isolated glomeruli and cultured mesangial cells as in vitro models to assess nephrotoxicity. Cell Biol Toxicol..

[25] Gómez-Guerrero C, Hernández-Vargas P, López-Franco O, Ortiz-Muñoz G, Egido J (2005). Mesangial cells and glomerular inflammation: from the pathogenesis to novel therapeutic approaches. Curr Drug Targets Inflamm Allergy..

[26] Satoh M, Kumar A, Kanwar YS, Reeves WH (1995). Anti-nuclear antibody production and immune-complex glomerulonephritis in BALB/c mice treated with pristane. Proc Natl Acad Sci U S A..

[27] Urbonaviciute V, Starke C, Pirschel W, Pohle S, Frey S, Daniel C (2013). Toll-like receptor 2 is required for autoantibody production and development of renal disease in pristane-induced lupus. Arthritis Rheum..

[28] Hou LF, He SJ, Li X, Wan CP, Yang Y, Zhang XH (2012). SM934 treated lupus-prone NZB × NZW F1 mice by enhancing macrophage interleukin-10 production and suppressing pathogenic T cell development. PLoS One..

[29] Bengtsson AA, Sturfelt G, Lood C, Rönnblom L, van Vollenhoven RF, Axelsson B (2012). Pharmacokinetics, tolerability, and preliminary efficacy of paquinimod (ABR-215757), a new quinoline-3-carboxamide derivative: studies in lupus-prone mice and a multicenter, randomized, double-blind, placebo-controlled, repeat-dose, dose-ranging study in patients with systemic lupus erythematosus. Arthritis Rheum..

[30] Oakley RH, Jewell CM, Yudt MR, Bofetiado DM, Cidlowski JA (1999). The dominant negative activity of the human glucocorticoid receptor beta isoform. Specificity and mechanisms of action. J Biol Chem.

[31] Rostami-Yazdi M, Clement B, Mrowietz U (2010). Pharmacokinetics of anti-psoriatic fumaric acid esters in psoriasis patients. Arch Dermatol Res.

[32] Scannevin RH, Chollate S, Jung MY, Shackett M, Patel H, Bista P (2012). Fumarates promote cytoprotection of central nervous system cells against oxidative stress via the nuclear factor (erythroid-derived 2)-like 2 pathway. J Pharmacol Exp Ther..

[33] Kakutani M, Takeuchi K, Waga I, Iwamura H, Wakitani K (1999). JTE-607, a novel inflammatory cytokine synthesis inhibitor without immunosuppression, protects from endotoxin shock in mice. Inflamm Res..

[34] Garrelds IM, van Hal PT, Haakmat RC, Hoogsteden HC, Saxena PR, Zijlstra FJ (1999). Time dependent production of cytokines and eicosanoids by human monocytic leukaemia U937 cells; effects of glucocorticosteroids. Mediators Inflamm..

[35] Bourke E, Moynagh PN (1999). Antiinflammatory effects of glucocorticoids in brain cells, independent of NF-kappa B. J Immunol..

[36] Fragaki K, Kileztky C, Trentesaux C, Zahm JM, Bajolet O, Johnson M (2006). Downregulation by a long-acting beta2-adrenergic receptor agonist and corticosteroid of Staphylococcus aureus-induced airway epithelial inflammatory mediator production. Am J Physiol Lung Cell Mol Physiol..

[37] Derijk RH, Schaaf MJ, Turner G, Datson NA, Vreugdenhil E, Cidlowski J (2001). A human glucocorticoid receptor gene variant that increases the stability of the glucocorticoid receptor beta-isoform mRNA is associated with rheumatoid arthritis. J Rheumatol..

[38] Honda M, Orii F, Ayabe T, Imai S, Ashida T, Obara T (2000). Expression of glucocorticoid receptor beta in lymphocytes of patients with glucocorticoid-resistant ulcerative colitis. Gastroenterology..

[39] de Zeeuw D, Akizawa T, Audhya P, Bakris GL, Chin M, Christ-Schmidt H (2013). Bardoxolone methyl in type 2 diabetes and stage 4 chronic kidney disease. N Engl J Med..

[40] Wu QQ, Wang Y, Senitko M, Meyer C, Wigley WC, Ferguson DA (2011). Bardoxolone methyl (BARD) ameliorates ischemic AKI and increases expression of protective genes Nrf2, PPARγ, and HO-1. Am J Physiol Renal Physiol.

[41] Wu T, Ye Y, Min SY, Zhu J, Khobahy E, Zhou J (2014). Prevention of murine lupus nephritis by targeting multiple signaling axes and oxidative stress using a synthetic triterpenoid. Arthritis Rheumatol..

[42] Tsai PY, Ka SM, Chang JM, Chen HC, Shui HA, Li CY (2011). Epigallocatechin-3-gallate prevents lupus nephritis development in mice via enhancing the Nrf2 antioxidant pathway and inhibiting NLRP3 inflammasome activation. Free Radic Biol Med..

[43] Takeda Y, Takeno M, Iwasaki M, Kobayashi H, Kirino Y, Ueda A (2004). Chemical induction of HO-1 suppresses lupus nephritis by reducing local iNOS expression and synthesis of anti-dsDNA antibody. Clin Exp Immunol..

[44] Calvani N, Caricchio R, Tucci M, Sobel ES, Silvestris F, Tartaglia P (2005). Induction of apoptosis by the hydrocarbon oil pristane: implications for pristane-induced lupus. J Immunol..

[45] Nacionales DC, Kelly KM, Lee PY, Zhuang H, Li Y, Weinstein JS (2006). Type I interferon production by tertiary lymphoid tissue developing in response to 2,6,10,14-tetramethyl-pentadecane (pristane). Am J Pathol..

[46] Chang A, Henderson SG, Brandt D, Liu N, Guttikonda R, Hsieh C (2011). In situ B cell-mediated immune responses and tubulointerstitial inflammation in human lupus nephritis. J Immunol..

[47] Aloisi F, Pujol-Borrell R (2006). Lymphoid neogenesis in chronic inflammatory diseases. Nat Rev Immunol..

[48] Wooley PH, Seibold JR, Whalen JD, Chapdelaine JM (1989). Pristane-induced arthritis. The immunologic and genetic features of an experimental murine model of autoimmune disease. Arthritis Rheum.

[49] Patten C, Bush K, Rioja I, Morgan R, Wooley P, Trill J (2004). Characterization of pristane-induced arthritis, a murine model of chronic disease: response to antirheumatic agents, expression of joint cytokines, and immunopathology. Arthritis Rheum..

[50] Lewis EJ, Hunsicker LG, Lan SP, Rohde RD, Lachin JM (1992). A controlled trial of plasmapheresis therapy in severe lupus nephritis. The Lupus Nephritis Collaborative Study Group. N Engl J Med.

[51] Rovin BH, Furie R, Latinis K, Looney RJ, Fervenza FC, Sanchez-Guerrero J (2012). Efficacy and safety of rituximab in patients with active proliferative lupus nephritis: the Lupus Nephritis Assessment with Rituximab study. Arthritis Rheum.

